# *Epichloë* Endophyte-Promoted Seed Pathogen Increases Host Grass Resistance Against Insect Herbivory

**DOI:** 10.3389/fmicb.2021.786619

**Published:** 2022-01-11

**Authors:** Miika Laihonen, Kari Saikkonen, Marjo Helander, Beatriz R. Vázquez de Aldana, Iñigo Zabalgogeazcoa, Benjamin Fuchs

**Affiliations:** ^1^Biodiversity Unit, University of Turku, Turku, Finland; ^2^Department of Biology, University of Turku, Turku, Finland; ^3^Institute of Natural Resources and Agrobiology of Salamanca (IRNASA-CSIC), Salamanca, Spain

**Keywords:** fungal endophyte, *Claviceps*, aphid, symbiosis, mutualism, herbivory, plant-microbe interactions, alkaloids

## Abstract

Plants host taxonomically and functionally complex communities of microbes. However, ecological studies on plant–microbe interactions rarely address the role of multiple co-occurring plant-associated microbes. Here, we contend that plant-associated microbes interact with each other and can have joint consequences for higher trophic levels. In this study we recorded the occurrence of the plant seed pathogenic fungus *Claviceps purpurea* and aphids (*Sitobion* sp.) on an established field experiment with red fescue (*Festuca rubra*) plants symbiotic to a seed transmitted endophytic fungus *Epichloë festucae* (E+) or non-symbiotic (E–). Both fungi are known to produce animal-toxic alkaloids. The study was conducted in a semi-natural setting, where E+ and E– plants from different origins (Spain and Northern Finland) were planted in a randomized design in a fenced common garden at Kevo Subarctic Research Station in Northern Finland. The results reveal that 45% of E+ plants were infected with *Claviceps* compared to 31% of E– plants. Uninfected plants had 4.5 times more aphids than *Claviceps* infected plants. By contrast, aphid infestation was unaffected by *Epichloë* symbiosis. *Claviceps* alkaloid concentrations correlated with a decrease in aphid numbers, which indicates their insect deterring features. These results show that plant mutualistic fungi can increase the infection probability of a pathogenic fungus, which then becomes beneficial to the plant by controlling herbivorous insects. Our study highlights the complexity and context dependency of species–species and multi-trophic interactions, thus challenging the labeling of species as plant mutualists or pathogens.

## Introduction

Plants, similar to all higher organisms, interact with an abundant and diverse microbiota, which is discovered to affect plant fitness (Zilber-Rosenberg and Rosenberg, 2008; Vandenkoornhuyse et al., 2015; Roughgarden et al., 2018; Enebe and Babalola, 2019; Saikkonen et al., 2020). Microbes, such as mycorrhizae, nitrogen-fixing bacteria, and asymptomatic endophytes, live symbiotically with their host plant. These symbionts can, for instance, affect the nutrient acquisition, phytohormone regulation, abiotic stress tolerance, and herbivory and pathogen resistance of their host (Ortíz-Castro et al., 2009; Berendsen et al., 2012; Dupont et al., 2015; Hassani et al., 2018; Nelson, 2018; Compant et al., 2019). Accordingly, these symbiotic microbes are commonly regarded as defensive plant mutualists, contrary to antagonistic parasitic and pathogenic microbes that have negative effects on host fitness (Clay, 2009).

However, the ecological roles of microbes in plant–microbe interactions are often complex, diverse, and prone to change from mutualistic to antagonistic and vice versa, depending on the abiotic and biotic conditions (Hayward, 1974; Carroll, 1988; Ahlholm et al., 2002; Partida-Martinez and Heil, 2011; Marsberg et al., 2017; Marchetto and Power, 2018; Shaffer et al., 2018; Meier and Hunter, 2019; Afkhami et al., 2020; Franklin et al., 2020; Laihonen et al., 2020; Petipas et al., 2020; Saikkonen et al., 2020). This continuum of antagonistic to mutualistic interactions in space and time (Saikkonen et al., 1998; Hirsch, 2004; Drew et al., 2021) must be considered in studies on plant microbe interactions. Furthermore, plants interact with myriads of organisms that probably interact not only with plants but also with each other (Lamichhane and Venturi, 2015; Bass et al., 2019). Despite the increasing number of studies characterizing plant microbiomes, ecological studies on the functional role of plant-associated microbes have only rarely examined the joint effects of the co-occurring microbes on the host plant. All this complexity of plant microbiomes shows the importance of comprehensive studies on reputed plant mutualistic or antagonistic microbes.

In this study, we examine the four-way interaction between red fescue (*Festuca rubra* L.), two fungal species, a vertically transmitted endophyte (*Epichloë festucae* Leuchtm., Schardl & M.R. Siegel) and a grass seed pathogen (*Claviceps purpurea* [Fr.] Tul.), and a phloem-feeding aphid (*Sitobion* sp.). *F. rubra* is a fine-leaved perennial grass growing in a wide range of habitats in the northern temperate zone. Some *Epichloë* species are common mutualistic symbionts of cool-season grasses. These systemic fungi grow asymptomatically in the aboveground parts of their hosts and reproduce asexually via host plant seeds (Schardl, 1996; Saikkonen et al., 2016). They often enhance their host's ability to endure abiotic stress, such as salinity or drought, or increase resistance against herbivores and pathogens (Schardl, 1996; Saikkonen et al., 1998, 2010). By contrast, *C. purpurea*, the ergot fungus, is widely recognized as a plant pathogen that infects the inflorescences in the Poaceae family. The fungus sterilizes the ovaries in flowers and utilizes the resources of its host to develop a sclerotium, preventing the development of a host plant seed. However, the pathogen usually castrates only a few flowers in a host plant inflorescence (Luttrell, 1980). Even though ecologically different, both *Epichloë* and *Claviceps* belong to the same fungal family, Clavicipitaceae.

Plant interactions with *Epichloë* and *Claviceps* have been thoroughly studied, but separately. Existing studies show that the presence of *Epichloë occultans* in *Lolium multiflorum* and *Epichloë gansuensis* in *Achnatherum inebrians* is correlated with a decreased frequency of *C. purpurea* infection, indicating that *Epichloë* mediates protection against *C. purpurea* (Pérez et al., 2013, 2017; Zhang et al., 2021). The increased pathogen resistance may result from the *Epichloë*-increased immunocompetence of the host, or from a direct competition between the invading microbe and the endophytic *Epichloë* (Saikkonen et al., 2016; Malinowski and Belesky, 2019). However, four-way interactions, including herbivores, have not been studied in this context. Both *Epichloë* and *Claviceps* produce animal-toxic alkaloids (Saikkonen et al., 2013; Miedaner and Geiger, 2015; Florea et al., 2017) that may synergistically increase herbivore resistance. The *Epichloë*-increased anti-herbivory defenses are primarily accounted for fungal-origin alkaloids including pyrrolizidines (lolines), ergot alkaloids, indolediterpenoids (lolitrems), and pyrrolopyrazines (peramine) (Saikkonen et al., 2013, 2016; Schardl et al., 2013). The sclerotia of *C. purpurea* contain high concentrations of ergot alkaloids that deter herbivores and fungivores (Miedaner and Geiger, 2015). Vertebrate herbivores avoid seed sets with only a few *C. purpurea* sclerotia which can increase host plant fitness in highly grazed environments. This attribute questions the strict pathogenic nature of *C. purpurea* (Wäli et al., 2013).

Here, in a four-way interaction, we examine how symbiotic *E. festucae* affects the natural colonization of *C. purpurea* and how these two fungi jointly affect herbivores in their common host plant in a semi-natural subarctic setting. We investigated in particular, whether:

1. The infection frequency of *Claviceps* differs between *Epichloë* symbiotic (E+) and *Epichloë* free (E–) plants
2. Aphid infestation on the plants is affected by either of the two plant-associated fungi
3. The chemical profile of ergot alkaloids can explain the infestation rate of aphids on the plants

All three species compete for the host plant's resources; however, we assume their ecological interactions with the host are as follows: *Epichloë* commonly forms mutualistic interaction with the host plant; *C. purpurea* is mainly antagonistic but potentially becomes beneficial under high herbivory pressure; and aphids as herbivores are pure antagonists to their host. Based on existing literature, we hypothesize that symbiosis with *Epichloë* decreases *Claviceps* infection frequency, and both fungal species contribute to plant defense, negatively affecting aphid performance. We discuss our findings regarding multiple contexts and defensive mutualism hypotheses.

## Materials and Methods

### Study System

The plants used in this study were part of a transplantation experiment designed to examine the importance of *E. festucae* in wild populations of red fescue (*F. rubra*) (Leinonen et al., 2019). We established identical experiments in three locations: Northern Finland; Southern Finland; and Salamanca, Spain. For this study, we utilized the Northern Finland experiment at Kevo Subarctic Research Institute (N 69.757 E 27.011, WGS84) to monitor naturally occurring herbivores and plant pathogens. The area was fenced to exclude large mammalian herbivores.

We planted *F. rubra* plants in a balanced and randomized common garden design in local sandy soil in 2018. Half of the plants originated from Northern Finland (in this study: “local plants”) and the other half came from inland Spain. The plants were collected from three Spanish and three Northern Finland populations (see details of locations in Leinonen et al., 2019). Half of the experimental plants harbored symbiotic *E. festucae* (E+), and the other half were endophyte-free (E–). A total of 120 plant individuals (genets) were divided into small ramets (clones) that were then planted. In the experiment, the plant clones were randomized to a block (12 ×10 plants), which was replicated five times (see Leinonen et al., 2019 for a detailed description of the entire setup). Thus, our experiment consisted of a total of 600 plants.

### Data Collection

#### Field Data

We recorded the occurrence of *C. purpurea* and the number of aphids on the experimental plants in their third growing season on August 12, 2020. We selected our timing according to two natural phenomena. First, *Claviceps*-infected plants were abundant that year in the region. Second, aphids were concentrated in inflorescences to feed on the nutrient-rich phloem, as flowering of the plants had just ended and seeds started to develop. This was the only time during our monitoring, since the establishment of the experimental field in 2018, when *Claviceps* infection and aphid infestation co-occurred on the experimental plants. As *Claviceps* infection visibly affects only the developing seeds that turn into sclerotia, we collected the data from all flowering individuals. We did not detect aphids feeding outside of the inflorescences, and we excluded non-flowering plant individuals from the data. We then identified the aphid species from photographs.

We seldom observed more than one *Claviceps* sclerotium in a single inflorescence, and thus we did not count the number of sclerotia per inflorescence. Our observation is in line with the literature stating that most host plant seeds develop normally (Luttrell, 1980).

#### Alkaloid Analyses

We analyzed ergot alkaloids from the inflorescences of 40 plants. All plants were of local origin. The selected plants evenly represented both endophyte-symbiotic (E+) and endophyte-free plants (E–), as well as *Claviceps*-infected (C+) and non-infected (C–) plants. The aphid distribution in the chosen plants appropriately represented the same pattern as in the original data. We clipped the plant inflorescences and stored them in deep freeze conditions until the analyses.

Plant samples were freeze dried and disrupted in a Mini-Beadbeater (Biospec Scientifica) and analyzed for ergot alkaloids following the procedure of Fuchs et al. (2013) with some modifications. In brief, samples (20 mg) were extracted with 150 μL of methanol and 150 μL of methylene chloride. After centrifugation two times for 10 min, the organic phases were combined. A 200-μL aliquot was evaporated, and the residue was dissolved in 25 μL of 80% methanol and analyzed using Liquid Chromatography Mass Spectrometry (LC/MS).

The analyses were performed using a UHPLC system (Agilent 1290 infinity II) coupled to a quadrupole time-of-flight mass spectrometer QTOF (Agilent G6546A). The separation of chemical compounds was conducted using a Zorbax Eclipse Plus C18 HD column (50 mm × 2.1 mm, 1.8 μm particle size; Agilent) at 30°C using the following solvents: solvent A consisted of aqueous formic acid (0.1%), and solvent B consisted of acetonitrile (100%). Gradient elution was performed using 100% B at a flow rate of 0.3 mL min^−1^ for 7 min and 100% B to 5% B in 3 min.

For the analysis of alkaloids, electrospray was operated in the positive ionization mode with the following settings: 225°C gas temperature, 13 L min^−1^ gas flow rate, 30 psgi nebulizer pressure, 350°C sheath gas temperature, and 7.5 L min^−1^ sheath gas flow. Ergot alkaloids (ergonovine, ergosine, ergotamine, ergocornine, α-ergocryptine, and ergocristine) were identified using the METLIN Metabolomics Database, and ergovaline was verified with a standard. All the alkaloids were quantified using ergotamine tartrate (Merck) as the standard.

#### Data Accessibility

The raw data supporting the conclusions of this article will be made available by the authors without undue reservation.

### Statistical Analyses

We ran all the analyses using the Statistical Analysis Software SAS 9.4. The FREQ procedure was used to describe the data. Given that several aphids on the plant were always present or not present at all, we conducted analyses for both the aphid number and the aphid presence/absence (binary variable).

We used the LOGISTIC procedure to conduct logistic regressions for *Claviceps* occurrence (C+ or C–) and aphid presence, with endophyte presence (E+ or E–), plant origin (Spain or local), genet, and block as explanatory variables. *Claviceps* occurrence was also an explanatory variable for aphid presence. We conducted a generalized linear model using the GLM procedure, in which the number of aphids was explained with *Claviceps* occurrence, plant's endophyte status and plant origin as fixed factors and block and genet as random factors. We used Tukey adjustment when comparing the aphid numbers between the groups.

Three E–C– alkaloid samples were removed from the analyses due to contamination. We used Student's *t*-tests (TTEST procedure) to compare the amounts of each distinguished ergot alkaloid in *Epichloë*-symbiotic and -free (E+, E–) plants and in *Claviceps*-infected and non-infected (C+, C–) plants. The GLM procedure with Tukey-adjusted *post-hoc* tests was used for pairwise comparisons among all four *Epichloë*–*Claviceps* combinatory plant groups (E–C–, E–C+, E+C–, and E+C+). Finally, we calculated Pearson correlation coefficients between the amounts of those alkaloids and aphid numbers in the sampled plants.

## Results

### Field Data

Our final field data resulted from a set of 176 flowering plants, since the rest of the plants did not produce inflorescences. A proportion of 46% of these plants were endophyte-free (E–). The majority of the plants were of local origin, as only 10 flowering plants (2 E– and 8 E+) were from Spain.

*Claviceps* infection was more common if the plant harbored the endophyte, occurring on 45% of the E+ plants compared with 31% of the E– plants (Table 1). *Claviceps* occurrence differed between the blocks [$X_{\left(4\right)}^{2}$ = 15.714, *p* = 0.003]; however, the interactions between the block and other variables were statistically non-significant.

**Table 1:** Summary of the fixed effects used in the models.

	**All plants**			**Local plants only**		
**Effect**	***X* ^2^**	***df***	***p***	***X* ^2^**	***df***	***p***
***Claviceps*** **occurrence**						
Endophyte	4.050	1	**0.044**	4.812	1	**0.028**
Plant origin	0.672	1	0.413			
Endophyte × plant origin	1.185	1	0.276			
**Aphid numbers**						
*Claviceps*	34.55	1,169	**<0.001**	35.47	1,159	**<0.001**
Endophyte	0.88	1,169	0.349	1.42	1,159	0.235
*Claviceps* × endophyte	0.16	1,168	0.685	0.38	1,158	0.537
Plant origin	2.25	1,168	0.135			
*Claviceps* × plant origin	1.00	1,167	0.319			
Endophyte × plant origin	0.08	1,167	0.775			
**Aphid presence**						
*Claviceps*	14.089	1	**<0.001**	13.649	1	**<0.001**
Endophyte	0.052	1	0.820	0.004	1	0.952
*Claviceps* × endophyte	1.734	1	0.188	2.056	1	0.152
Plant origin	0.708	1	0.400			
*Claviceps* × plant origin	0.045	1	0.833			
Endophyte × plant origin	0.002	1	0.968			

*All analyses were conducted for all plants and for local plants. Endophyte affected the occurrence of Claviceps, and Claviceps affected both aphid numbers and presence. Statistically significant (<0.05) p-values are bolded. N_all plants_ = 176; N_local plants_ = 166*.

**Figure 1:**
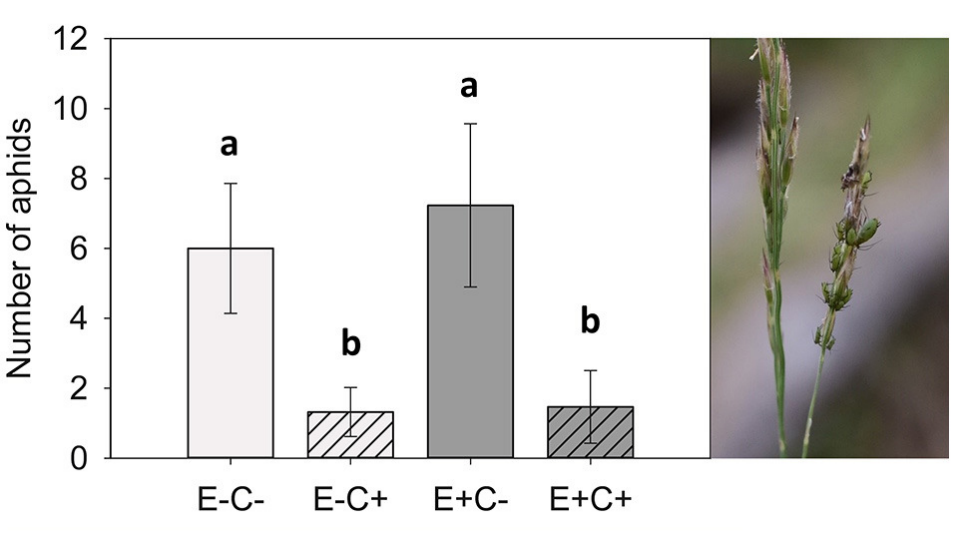
Number of aphids on the study plants. There were over 4.5 times more aphids on the plants without the *Claviceps* (C) infection. *Epichloë* (E) did not affect the amount of aphids on the plant. Generalized linear model, Tukey-adjusted pairwise comparisons: E–C– and E–C+: t_167_ = 3.48, *p* = 0.004; E–C– and E+C–: t_167_ = 1.13, *p* = 0.670; E–C– and E+C+: t_167_ = 3.93, *p* = 0.001; E–C+ and E+C–: t_167_ = 4.36, *p* = < 0.001; E–C+ and E+C+: t_167_ = 0.24, *p* = 0.995; E+C– and E+C+: t_167_ = 4.92, *p* = < 0.001. Error bars represent 95% confidence intervals. *N* = 176.

**Figure 2:**
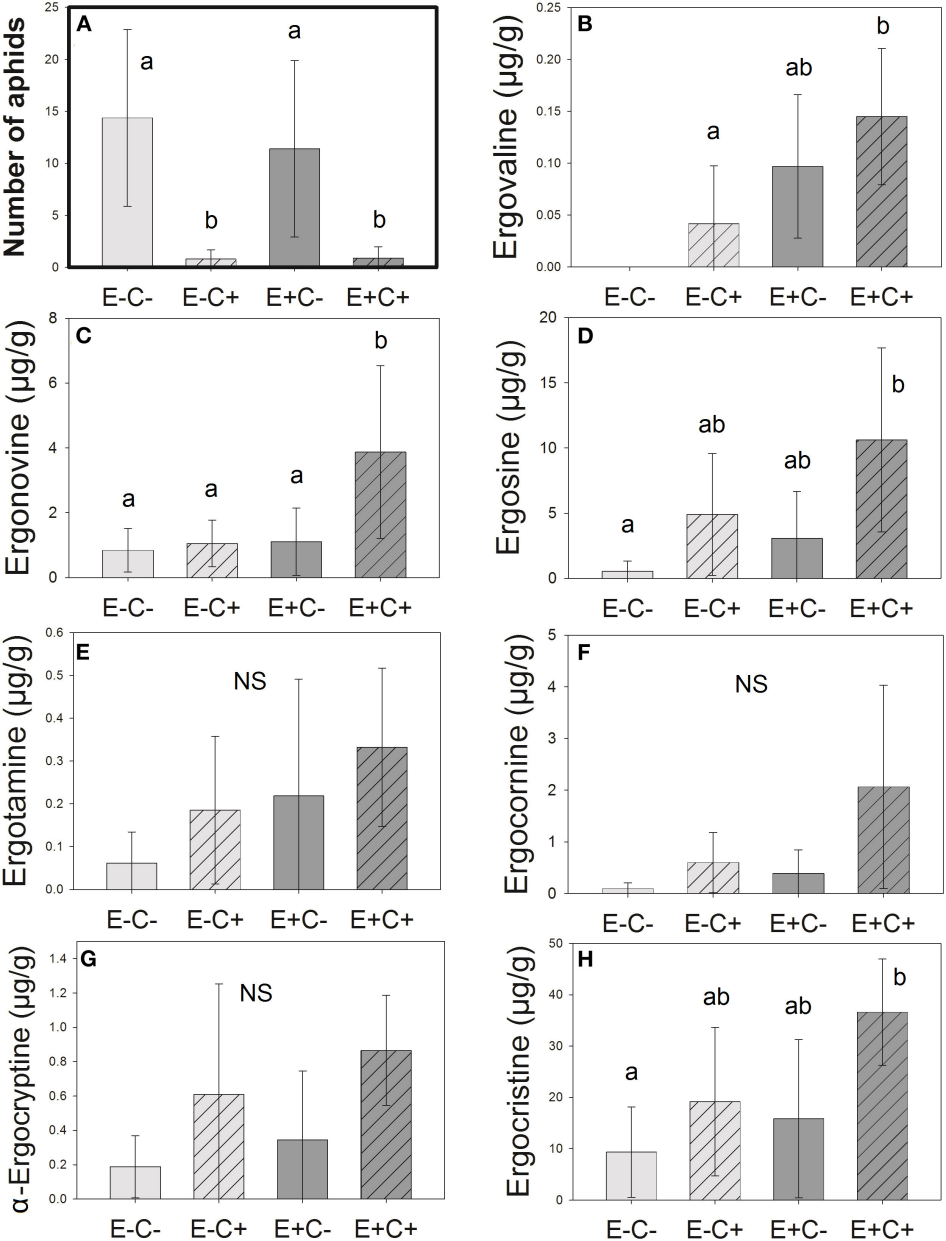
Number of aphids **(A)** next to the ergot alkaloid concentrations **(B–H)** in the samples analyzed. Alkaloid concentrations tend to be the highest in plants that are both *Epichloë*-symbiotic and *Claviceps*-infected (E+C+). Letters represent statistically significant differences (*p* < 0.05, Tukey adjusted). Error bars represent 95% confidence intervals. *N* = 37.

Aphid numbers were 4.5 times higher in non-infected plants (C–) than in plants infected with *Claviceps* (C+). However, aphid numbers did not differ between endophyte-symbiotic (E+) and endophyte-free (E–) plants (Table 1 and Figure 1). The plants selected for chemical analysis represented the same pattern (Figure 2A). Regarding presence data, aphids were not found in 59% of the *Claviceps*-infected (C+) plants compared with 29% of the non-infected (C–) plants [Table 1; block: $X_{\left(4\right)}^{2}$ = 28.326, *p* = < 0.001, interactions with block non-significant].

The genet and all interactions between the explanatory variables were not statistically significant and were removed from the final models.

As almost 95% of the plants within the final dataset were of local origin, we also ran the analyses exclusively for the local-origin plants. The results were similar to those presented above (Table 1).

Initially, we ascribed the aphids to a single species in the genus *Sitobion*. Aphid specialist Anders Albrecht (Finnish Museum of Natural History) confirmed that they were most likely *Sitobion avenae* (Fabricius, 1775), although *Sitobion fragariae* (Walker, 1848) could not be completely ruled out based on identification from photographs (Figure 1).

### Alkaloid Analyses

We identified seven different ergot alkaloids in plant extracts: ergovaline, ergonovine, ergosine, ergotamine, ergocornine, α-ergocryptine, and ergocristine. Highest ergovaline concentrations were detected in inflorescences of *Epichloë-*symbiotic (E+) plants, and its concentrations correlated negatively with aphid numbers (Table 2). We found ergosine, ergocornine, α-ergocryptine, and ergocristine mainly in inflorescences having sclerotia of *Claviceps* (C+). Ergonovine concentrations were associated with endophytic *Epichloë*, but association with *Claviceps* was also marginally significant. We could not associate ergotamine with either fungi. The concentrations of ergovaline, ergotamine, and ergocristine correlated negatively with aphid numbers. However, correlations with other alkaloids were also close to statistical significance (Table 2).

**Table 2:** Analysis of ergot alkaloids showing which alkaloids were associated with *Epichloë* symbiosis and visible *Claviceps* infection and how their concentration correlated with the number of aphids in the same plants.

		**Ergovaline**	**Ergonovine**	**Ergosine**	**Ergotamine**	**Ergocornine**	**α-Ergocryptine**	**Ergocristine**
*Epichloë*	t	3.58	2.10	1.54	1.53	1.66	0.75	1.67
	*df*	35	22.6	35	35	23.3	35	35
	*p*	**0.001**	**0.047**	0.132	0.135	0.111	0.457	0.105
*Claviceps*	t	−1.18	−1.99	−2.63	−1.11	−2.17	−2.25	−2.32
	*df*	35	25.5	27.8	35	21.5	35	35
	*p*	0.247	0.058	**0.014**	0.276	**0.042**	**0.031**	**0.026**
Aphids	*r*	−0.325	−0.302	−0.309	−0.338	−0.243	−0.319	−0.405
	*p*	**0.049**	0.069	0.062	**0.041**	0.147	0.054	**0.013**

*Statistically significant (<0.05) p-values are bolded. N = 37*.

Some seemingly uninfected plants contained low alkaloid amounts (Figures 2B–H). This finding is probably due to the emerging, still invisible *Claviceps* infections or sporal contaminations. *Epichloë*-symbiotic plants that were also *Claviceps* infected (E+C+) had significantly higher concentrations of ergonovine than other plants (Figure 2C). Concentrations of ergovaline were higher in the E+C+ plants than in the E–C– or E–C+ plants (Figure 2B), and the concentrations of ergosine and ergocristine were significantly higher in E+C+ than those in the E–C– (Figures 2D,H). We did not find statistically significant differences in the pairwise comparisons between any *Epichloë*–*Claviceps* combinatory plant groups in ergotamine, ergocornine, and α-ergocryptine concentrations (Figures 2E–G).

## Discussion

Our results do not support the hypothesis that defensive mutualism against invertebrate herbivores and pathogens drives endophyte–plant symbiosis. Contrary to our predictions that *E. festucae* should increase the resistance of its host grass, *F. rubra*, the aphid numbers were similar on *Epichloë*-symbiotic (E+) and *Epichloë*-free (E–) plants, and the infection frequencies of pathogenic *Claviceps* were higher in E+ plants than in E– ones. However, as expected, *Claviceps* provided notable protection against aphid herbivory, suggesting that the net effects on the host can remain positive as the infection causes only minor seed loss (Luttrell, 1980; Wäli et al., 2013). These results emphasize the importance of understanding the structure and functional complexity of plant microbiomes by demonstrating that plant–pathogen interactions are context dependent, ranging from antagonistic to mutualistic, rather than always disadvantageous to the host plant (Saikkonen et al., 1998, 2020; Vázquez de Aldana et al., 2010; Wäli et al., 2013; Rybakova et al., 2016; Selosse et al., 2018).

As the defensive mutualism between grasses and *Epichloë* species is primarily attributable to fungal origin alkaloids (Saikkonen et al., 2010; Schardl et al., 2013), our results suggest that the fungal genotypes associated with the plants could not provide protection against aphids by producing a sufficient amount of alkaloids. Our previous transplant study with *F. rubra* plants collected from the same wild populations revealed that *E. festucae* can produce peramine and ergovaline; however, alkaloid profiles in plants varied among the geographic origin and growth conditions of the symbiotum (Vázquez de Aldana et al., 2020). Peramine was only produced by the Spanish genotypes. Ergovaline was detected in E+ plants across Europe, but the concentrations varied among fungal strains, and the profiles changed when the symbiotum was transplanted to new environments. For example, none of the Spanish E+ plants contained detectable amounts of ergovaline in Northern Finland. In line with our previous study, ergot alkaloid concentrations varied considerably among E+ plants in the present study. Similarly, many other studies with other grass species have revealed that the quantities of *Epichloë* alkaloids vary among geographic origin and genetic plant–fungus combinations and can be plastic during the growing season depending on environmental conditions (Siegel et al., 1990; Thom et al., 2014; Helander et al., 2016; König et al., 2018; Fuchs et al., 2020).

In this study, we focused on the putative anti-herbivore properties of ergot alkaloids, which are a diverse family of mycotoxin compounds with a common origin (Panaccione, 2005). Unlike the majority of past studies treating ergot alkaloids as a pooled ensemble, we identified seven different ergot alkaloids from plant extracts (ergovaline, ergonovine, ergosine, ergotamine, ergocornine, α-ergocryptine, and ergocristine) and examined their potential in modulating host plant quality to aphids. Ergovaline is the main ergot alkaloid produced by *Epichloë* in most host grasses and a minor component of *C. purpurea* (Garner et al., 1993); the other six compounds are the main alkaloids of *C. purpurea* (Miedaner and Geiger, 2015). Our alkaloid results are in line with this literature, but ergonovine was also present in E+ plants, and we were not able to clearly associate presence of ergotamine with *Claviceps* infection only. The few earlier studies on individual alkaloids have shown variable effects on herbivores. For example, ergonovine is responsible for aphid mortality on sleepygrass (*Achnatherum robustum*) (Shymanovich et al., 2015), and similarly ergocryptine markedly explains the variation in fall armyworm dry weight on perennial ryegrass (*Lolium perenne*) (Salminen et al., 2005). By contrast, ergovaline appears to be insignificant for the performance of root or shoot aphids (Siegel et al., 1990; Popay et al., 2021). The issue becomes even more complicated when other trophic layers are involved. For example, Kunkel et al. (2004) showed that ergot alkaloids can mediate cascading effects in food webs, thereby indirectly benefiting herbivores due to their toxic effects on their natural enemies. In concordance with these studies, our results demonstrate dissimilar effects of individual ergot alkaloids on herbivores. Overall, the concentrations of all examined alkaloids were highest in *Claviceps*-infected E+ plants, suggesting the synergistic effect of both fungi on the alkaloid profile of the host grass. Concentrations of three out of the seven examined alkaloids (ergovaline, ergotamine, and ergocristine) were negatively correlated with the number of aphids on the plants. *Epichloë* symbiosis explained only the presence of ergovaline and ergonovine, whereas *Claviceps* explained the concentrations of ergosine, ergocornine, α-ergocryptine, and ergocristine. Furthermore, ergocristine was associated with both *Claviceps* infection and the low number of aphids on the plants. We acknowledge that the chemical ecology underlying these results should be interpreted cautiously, since we studied only ergot alkaloids, and the analyses do not allow us to distinguish other *Epichloë*- and *Claviceps*-origin alkaloids in the samples. Still, since we found no compelling evidence that *Epichloë* symbiosis confers protection against aphids, we suggest that *Claviceps* is primarily responsible for the production of anti-herbivore compounds and for the reduction of aphid infestation. However, we acknowledge that additional chemical changes may occur (volatiles, etc.), which when combined with the examined alkaloids might reduce the aphid infestation in C+ plants.

The lack of *Epichloë*-enhanced host grass resistance to aphids can be partly explained by the high variation among the examined fungal lineages in their ability to produce alkaloids due to their genetic and chemotypic differentiation in Spanish and Finnish plants under different selection pressures. Empirical evidence supports the idea that post-glacial colonization history and contrasting climatic environments have resulted in local adaptations and genetic differentiation in symbiotum across its range in Europe (Dirihan et al., 2016; Leinonen et al., 2019; von Cräutlein et al., 2019, 2021). Strong seasonal changes in temperature, including short growing seasons and long winters, and variation in day length and light quality characterize environments at high latitudes, whereas plants in Spanish semiarid grasslands must adapt to seasonal drought (Zabalgogeazcoa et al., 2006; Leinonen et al., 2019). Genetic potential for diverse alkaloid production is remarkable in southern populations due to the prevalence of sexual reproduction in Spanish *Epichloë* populations (von Cräutlein et al., 2019, 2021). Although the northern populations have not adapted to strong invertebrate herbivory, the typically high *Epichloë* frequencies in wild *F. rubra* populations across its range in Europe are likely to be attributable to other benefits associated with *Epichloë*. Our previous transplant experiment with plants collected from the same geographic regions suggests that abiotic factors have not played a significant role in maintaining *Epichloë* symbiosis (Dirihan et al., 2016; Leinonen et al., 2019). Many examined European *F. rubra* populations with high frequencies of E+ commonly show strong vertebrate grazing—for example, by sheep in the Faroe Islands and Iceland and large ungulates in Spain, Switzerland, and northern Finland (Dirihan et al., 2016). This finding suggests that vertebrate grazing has been among the main selective forces driving the coevolution of *E. festucae* and *F. rubra* in Europe.

Increased susceptibility of E+ plants to the pathogenic *Claviceps* fungus compared with their E-conspecifics suggests that reciprocal changes during the long coevolutionary history of *Epichloë*-species and their host grasses involve the loss of host traits that prevent microbial invasions. These changes may result from modulated recognition, signaling, and defense responses (Saikkonen et al., 2013; van Overbeek and Saikkonen, 2016; Schmid et al., 2017; Bastías et al., 2018; Compant et al., 2019; Nissinen et al., 2019). Our results demonstrate that the consequences of such a predisposition to pathogens can be advantageous to the host plant in environments where a pathogen with only marginal damage to the host provides reinforced protection against pests. This finding questions whether these changes in host traits resulted from tripartite coevolution with reciprocal changes in the partners.

## Conclusions

We propose that the independent coevolution of these two closely related fungi with their shared host plant might have resulted in the detection of complementary protection against herbivores and other benefits to each other as by-products without reciprocity and cooperation (Leimar and Connor, 2003). Vertical transmission and alkaloid production ability of *Epichloë*-species have selected benign symbiosis with the host grass, particularly in environments driven by strong herbivory pressure. The fitness of *Claviceps* is similarly highly dependent on the protection of host inflorescences to ensure successful sclerotium development to complete its life cycle. However, the occurrence and distribution of *Claviceps* is primarily dependent on the presence of the host and favorable weather and climatic conditions, whereas the heritable infections of *Epichloë* species depend on the host fitness. Although host protection can be regarded as an indirect by-product of mutualism, our results are consistent with a few other recent findings, suggesting that *Epichloë* species must be considered keystone species in shaping the microbial communities of their shared hosts (Nissinen et al., 2019).

## Data Availability Statement

The raw data supporting the conclusions of this article will be made available by the authors, without undue reservation.

## Author Contributions

ML and BF originally formulated the idea, performed the experiments, and analyzed the data. ML, BF, KS, and MH conceived and designed the experiments. BV and IZ performed the chemical analyses. ML, KS, MH, BV, IZ, and BF wrote the manuscript. All authors contributed to the article and approved the submitted version.

## Funding

This work was supported by the Academy of Finland (KS, Grant Nos. 295976 and 326226; MH, Grant No. 311077; BF, Grant No. 324523), the Spanish Ministry of Science and Innovation and FEDER grant PID2019-109133RB-I00, and from the project CLU-2019-05—IRNASA/CSIC Unit of Excellence funded by Junta de Castilla y León and co-financed by the European Union (ERDF Europe drives our growth).

## Conflict of Interest

The authors declare that the research was conducted in the absence of any commercial or financial relationships that could be construed as a potential conflict of interest.

## Publisher's Note

All claims expressed in this article are solely those of the authors and do not necessarily represent those of their affiliated organizations, or those of the publisher, the editors and the reviewers. Any product that may be evaluated in this article, or claim that may be made by its manufacturer, is not guaranteed or endorsed by the publisher.
